# Supplementary material for: Process Control Monitor (PCM) for Simultaneous Determination of the Piezoelectric Coefficients d31 and d33 of AlN and AlScN Thin Films
Source: Micromachines (Basel). 2022 Apr 7;13(4):581. doi: 10.3390/mi13040581 (PMC9030765; doi:10.3390/mi13040581)
Supplement: Supplementary file 1 [file micromachines-13-00581-s001.zip › micromachines-1644013-supplementary.pdf]

Experimental detail

The LDV test was applied on Polytec MSA 500, a 65 kHz AC excitation is amplified to 100 V by a high voltage amplifier (Aigtek ATA-2032), and applied to the electrode. Since the test structure is a complete bulk structure, the resonance frequency obtained is on the order of GHz. Therefore, we took a square electrode with a center electrode width of 500 μm and an outer electrode width of 125 μm as examples. The test frequency is far from the resonance point, so there is no possibility of resonance amplification. We used LDV to conduct multiple tests, and the test results have good repeatability. In this work, only one test results was shown as the fitting comparison data.

The DBLI-based method was carried out on Aixacct Aix DBLI. The top and bottom beams of DBLI were projected on the central electrode on the upper surface and the lower surface of the Si substrate respectively. The test is at 1000 Hz and 100 V AC voltage.

The bottom electrode of the test samples used in in-situ XRD measurement was grounded, and DC voltage was applied to the top electrode. DC voltages of -50 V, -30 V, 0 V, 30 V and 50 V were applied to the samples for five cycles of testing.

The cantilever test method uses the same test system as LDV test. A 500 Hz AC excitation was amplified to 50 V and applied to the top and bottom electrodes of cantilevers. The cantilevers resonant frequencies are on the order of hundreds of kHz. The test frequency is far from the resonance point, so the test results are not affected by resonance amplification.
